# Modeling voxel-based Monte Carlo light transport with curved and oblique boundary surfaces

**DOI:** 10.1117/1.JBO.25.2.025001

**Published:** 2020-02-25

**Authors:** Anh Phong Tran, Steven L. Jacques

**Affiliations:** a Northeastern University, Department of Chemical Engineering, Boston, Massachusetts, United States; b University of Washington, Department of Bioengineering, Seattle, Washington, United States

**Keywords:** 3D photon transport, curved boundaries, Fresnel laws

## Abstract

**Significance:** Monte Carlo (MC) light transport simulations are most often performed in regularly spaced three-dimensional voxels, a type of data representation that naturally struggles to represent boundary surfaces with curvature and oblique angles. Not accounting properly for such boundaries with an index of refractivity, mismatches can lead to important inaccuracies, not only in the calculated angles of reflection and transmission but also in the amount of light that transmits through or reflects from these mismatched boundary surfaces.

**Aim:** A new MC light transport algorithm is introduced to deal with curvature and oblique angles of incidence when simulated photons encounter mismatched boundary surfaces.

**Approach:** The core of the proposed algorithm applies the efficient preprocessing step of calculating a gradient map of the mismatched boundaries, a smoothing step on this calculated 3D vector field to remove surface roughness due to discretization and an interpolation scheme to improve the handling of curvature.

**Results:** Through simulations of light hitting the side of a sphere and going through a lens, the agreement of this approach with analytical solutions is shown to be strong.

**Conclusions:** The MC method introduced here has the advantage of requiring only slight implementation changes from the current state-of-the-art to accurately simulate mismatched boundaries and readily exploit the acceleration of general-purpose graphics processing units. A code implementation, mcxyzn, is made available and maintained at https://omlc.org/software/mc/mcxyzn/.

## 1 Introduction

The propagation of photons in a Monte Carlo (MC) simulation can be broken down in a few standard steps: propagation (hop), absorption (drop), scattering (spin), and the Fresnel phenomena of transmission and reflection at boundaries, where the refractive index changes.^1^ When using a rectangular grid structure, the representation of curved, smooth, or slanted boundaries is discretized into voxels, also referred to as a Cartesian grid.^2^ The drawbacks are not only aesthetic in nature but also represent important deviations from the modeled structure. The use of a finer grid can partially minimize the differences between the voxel-based representation and the modeled geometry. This works particularly well to improve the precision of the propagation, absorption, and scattering steps; the trade-off being higher memory requirements to store the information and slower simulation performances due to the larger number of elements involved. The deviation addressed in this work is the representation of the surface boundary between mismatched materials, having different indices of refractivity, being simplified to the facets of the voxels located at the boundary. No matter how small the voxels, the normal angles to those boundary facets are intrinsically limited to being one of the facets of these voxels. This is of particular importance when dealing with reflection and transmission using Fresnel equations because the normal angle to the surface not only plays a key role in determining the amount of light that is to be reflected or transmitted but also determines the altered angles for the trajectories of these photons during the propagation process.

This problem of correctly modeling the curvature or orientation of the object geometry has been a recurrent theme in the MC simulation of light transport literature.^2^^,^^3^ Efforts to address this issue have been generally categorized into three methodologies: tetrahedron-based MC, geometry-based MC, and surface-based MC.^3^ Tetrahedron-based MC addresses the problem by creating a labeled tetrahedral mesh of the voxelated volume. The propagation is very similar to voxel-based MC except that the curvature on surface boundaries can be better approximated through triangular surfaces. The usual drawbacks are the challenges and efforts associated with creating a high-quality tetrahedral mesh,^4^ but also that different mesh generation algorithms will produce different mesh volumes that may not correspond well with the starting volume as well as requiring more complex ray tracing of the photons. This is currently the preferred method when dealing with this problem with popular software, such as TIM-OS,^5^ MMC,^6^ and recently, a GPU-accelerated version of this approach, FullMonteCUDA.^7^ A hybrid methodology using tetrahedron-based MC but saving the outputs inside a voxelated grid was also proposed.^8^ When the geometry can be well described mathematically, geometry-based MC has been proposed^9^^–^^13^ with the limitation that it is very problem-specific and lacks versatility to address the usual complexity of biological tissues. This is mainly because complex tissue structures need to be approximated by geometries for which an analytical boundary surface can be derived, such as a sphere, an ellipse, or a cylinder. The last approach, surface-based MC, combines a surface, usually triangular, of the mismatched boundaries and the usual voxel-based propagation algorithm.^14^^,^^15^ The propagation volume and saved fluence volume are the same as the voxel-based approach, but at each propagation step, there is an additional calculation that checks and handles the possible crossing of a mismatched boundary surface. The generation and handling of this boundary surface to calculate reflection and refraction is very similar to the tetrahedron-based MC methods. This was also translated in a GPU-accelerated environment.^16^

The objective of this work is to preserve most of the core voxel-based MC algorithm that was outlined in earlier work^17^^–^^19^ as well as exploiting the simulation speedup acceleration brought by the use of multicore and GPGPU architectures,^20^^,^^21^ while also addressing this issue of curvature and slanted surface boundaries. The implementation of this algorithm was based on that of mcxyz.c.^22^

## 2 Materials and Methods

When a photon propagates and traverses a boundary with mismatched indices of refractivity, it needs to decide whether to reflect or to transmit. The angles of reflectance and transmittance and the corresponding event probabilities are determined through Fresnel equations using the angle of incidence as well as the normal angle to the boundary surface. If these angles are not handled properly, significant problems will appear when dealing with light sources shining at an angle with respect to the nonflat target or when encountering curvature.

### 2.1 Normal Vector Estimation and Smoothing

In an effort to better estimate the direction of the mismatched surface, an edge detection algorithm, the Sobel filter,^23^ is applied to create a gradient map. By applying such filters to a 3D map with each voxel containing its respective index of refraction, not only can the edges be calculated but also the direction of change is specified. In addition, by applying a Sobel–Feldman type operator, an additional smoothing effect is observed. More sophisticated algorithms to better approximate the normal vectors to the surface certainly exist, but this report is limited to investigating the simple 3×3×3 Sobel filter to illustrate the usefulness of this approach and highlight the low computing cost of this additional step. In the 2D case, the Sobel operator can reduce from a maximum angle error of 45 deg using the facet normal of a voxel down to an estimated maximum error value of 1.36 deg.^24^ The 3×3×3 Sobel filter is readily available in MATLAB through the function imgradientxyz.

**Fig. 1:**
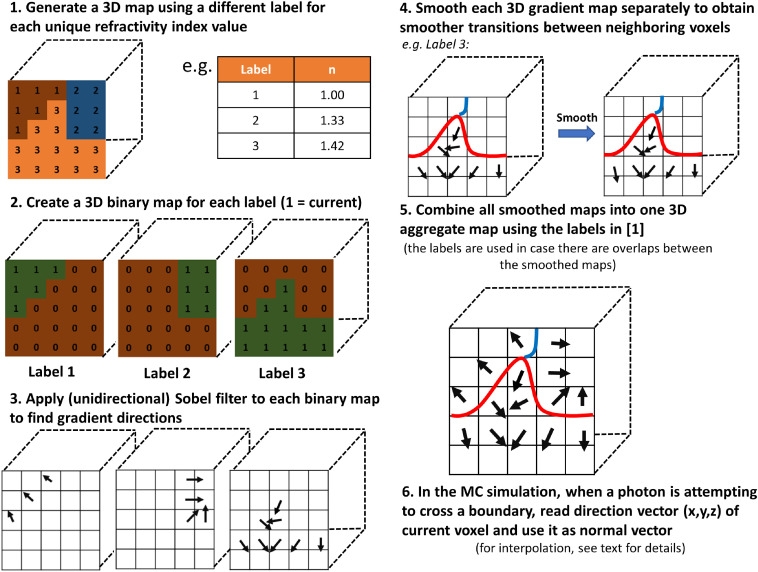
Proposed algorithm to handle curvature and oblique angles in MC light simulations.

Figure 1 illustrates the method for specifying the boundaries and the unit vectors that are normal to that surface in each voxel containing the surface. An expanded explanation containing the implementation details is available in Sec. 5. The steps are (following the order of Fig. 1) as follows.

1. Create a labeled 3D map for the refractive indices (n). The example shows a 3D map with label values 1, 2, and 3, each used to represent one of the three unique values of n. This map is used to delineate the mismatched boundaries, where the Fresnel equations are applied.
2. Create a 3D binary map for each of the labels in step 1 (or equivalently, for each unique refractive index value), labeling a voxel 1 if it matches the label of the map and 0 otherwise. The example for step 2 shows three such binary maps for each of the labels in step 1.
3. Apply the MATLAB Sobel filter using imgradientxyz to each binary map in step 2. This will yield the direction vectors contained inside of each of the labeled regions. These direction vectors are normal to the mismatched boundaries in 3D (though represented in 2D in the example for simplicity).
4. Smooth each of the 3D gradient map separately in step 3, which is important to ensure that vectors on opposite sides of a mismatched boundary do not interfere with one another (for example, by canceling each other out). In this work, smoothn^25^^,^^26^ was used to smooth each of the gradient maps. Visual feedback is recommended when attempting to determine the appropriate smoothing factor in smoothn for a given problem. A value chosen to be too high will likely deviate too much from the starting geometry.
5. Combine all the smoothed maps in step 4 into one aggregate 3D map with all the direction vectors. The smoothing will likely lead to some overlaps between the 3D gradient maps. In order to resolve this issue, each direction vector element of a smoothed 3D gradient map is multiplied by its corresponding element (0 or 1) in the 3D binary map of the same label (calculated in step 2). This operation removes the direction vectors in the smoothed gradient maps (by making them zero vectors) at the voxel locations, where the 3D binary map of the same label or refractive index is 0. Once this is done, one simply sums these maps together into an aggregate 3D map like the one shown in the illustrated example.
6. The final step is to normalize all the direction vectors and to store these direction vectors in such a way that they can be used as normal vectors in the Fresnel equations when a photon is attempting to cross a mismatched boundary during the MC simulation.

The justification for the smoothing is that when calculating direction vectors, the Sobel filter is local in scope and suffers from the staircase pattern that forms when discretizing into a regular grid. Thus, the smoothing helps the algorithm to estimate curvature more realistically as it gives a dependence of each direction vector on its neighbors.

These normal vectors that are calculated near the edges, where a refractive index mismatch occurs, are then saved into a storage matrix that can be consulted by the MC software. In Fig. 1, to illustrate the concept, the x, y, and z directions of the normal vectors for each voxel of the simulation domain were saved into three 3D cubes. The implementation of the gradient calculations and smoothing steps was made in MATLAB and the smoothed gradient map was then saved in a binary file that was subsequently read by mcxyzn. It is important to note that this map is only consulted when a photon is about to cross a boundary with a refractive index mismatch, thus, it only alters the performance of the MC software when this normal vector needs to be read from memory. If memory is an issue, it is trivial to create a 3D hash table, a data structure that maps keys to values, that would be associated with a matrix that would only store the direction vectors for the nonzero vectors. Each entry of the hash table would give the index (or key) corresponding to the direction vector in the look-up table. As a photon crosses a boundary into a new voxel with a different refractive index, the hash-table pointer of the new voxel would point to the hash table to find the surface normal vector. The benefit being that the 3D storage of one key value at each voxel uses less memory than having to store a triplet of vector values at each location.

### 2.2 Level of Details in Calculating Fresnel Equations

**Fig. 2:**
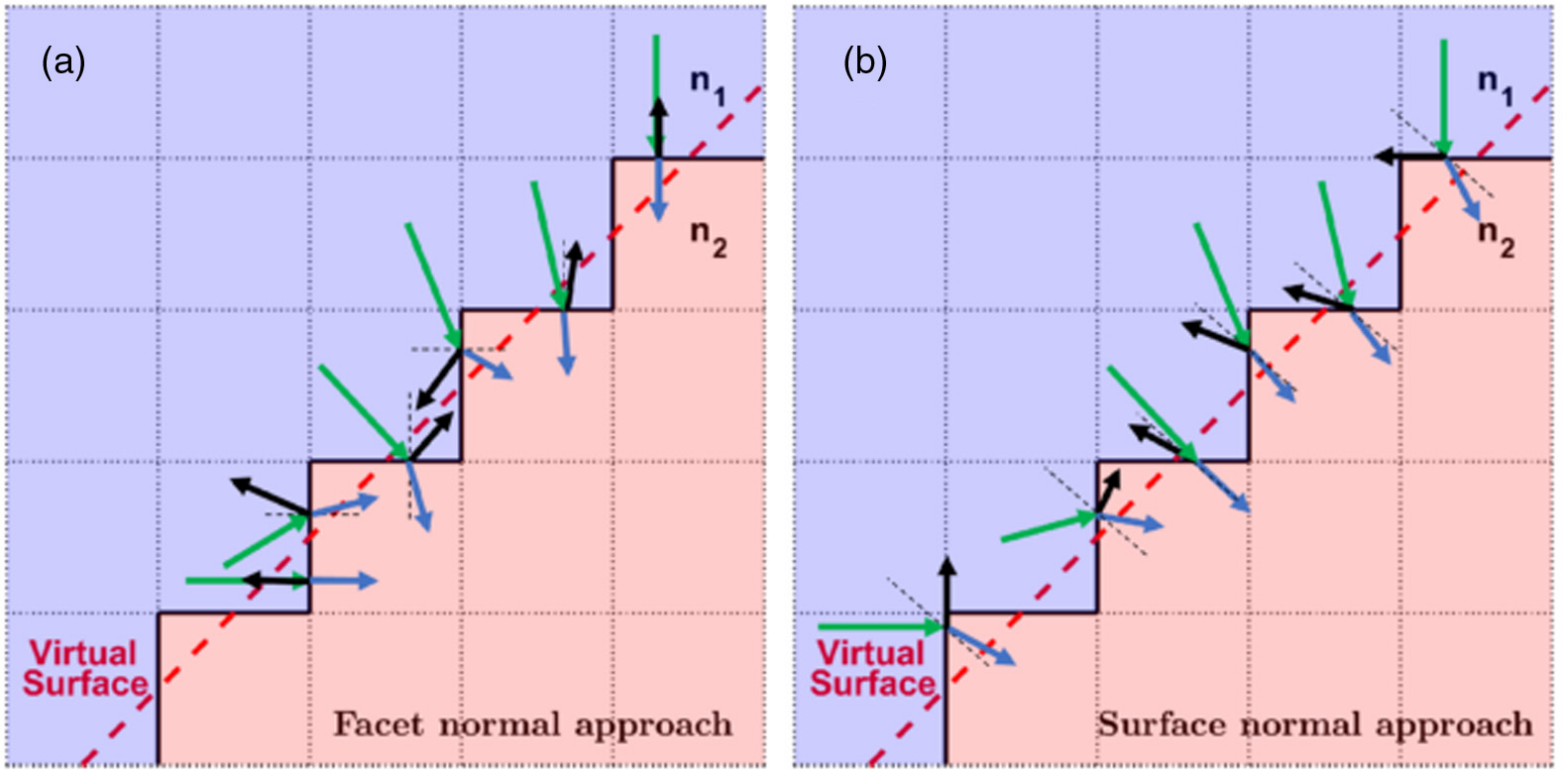
Using virtual surface that lies at a 45 deg angle for simplicity, various transmission and reflectance examples are shown for (a) the facet normal approach and (b) the surface normal approach. In the facet normal approach, small variations in the angle of incidence at which a photon hits a mismatched boundary can lead to dramatic changes in the reflected and transmitted angles. Using very similar scenarios under the surface normal approach corrects for these errors in angles.

In Fig. 2, we show two methods: the facet normal approach, where only the normal vectors to the facets of voxels are considered, and the surface normal approach, where the normal estimate to the mismatched boundary is being used in the propagation of photons. It is important to note that the facet normal approach can lead to significant errors in the angles of transmittance and reflectance but also can create large discrepancies in the proportion of light that transmits versus reflects at the mismatched boundaries. The surface normal approach reduces these errors significantly.

When a photon is at the mismatched boundary, simply using the normal gradient calculated from the Sobel filter already represents a significant improvement over the facet normal approach. A downside of this approach is that when modeling a curvature in a low-scattering medium (e.g., light “bouncing off” the surface of a spherical object in water), the change in angle direction is still discrete in nature and not gradual as one might expect. To further enhance the proposed algorithm, the interpolated surface normal approach is introduced in the next section to deal with curvature.

### 2.3 Intermediate Angle Interpolation Approach

To model gradual changes in normal angles, the use of the trilinear interpolation^27^^,^^28^ is introduced. The basic method interpolates the values of gradient at the eight voxels surrounding the photon, applying separately to the x, y, and z gradients. In the modification used in this work, the interpolation only considers the voxel gradients on the side of the surface from which the photon is propagating. The interpolated normal vector is then normalized. The details on how to apply this interpolation are provided in Sec. 5.2.

This interpolated normal approach has a higher computational cost than the simple surface normal approach in the previous section. Instead of having to read the values of one direction vector, the interpolated surface normal algorithm is now required to read the gradient direction values of the eight closest neighbors to this intersection point as well as their respective index of refractivity, and then apply the interpolation scheme to these values.

## 3 Results and Discussion

Three examples showcase the strengths and weaknesses of the proposed method: (1) a beam hitting a sphere surface, (2) light transmission through a lens, and (3) light hitting the forehead of a mouse. In the first two cases, simulations not involving scattering are shown and compared to the analytical solutions for which the expected behavior is known. The reported simulation speeds are for a GPU-accelerated OpenCL implementation of mcxyzn running on a NVIDIA RTX 2080 graphics card and an i7-7820X Intel processor (8 cores and 16 threads). The fluence rate φ has the units of $W/{\mathrm{cm}}^{2}/W$ delivered, assuming a source power of 1 W. This normalized quantity can be scaled proportionally to the amount of W being delivered by the source.

### 3.1 Laser Beam Hitting the Surface of a Sphere

**Fig. 3:**
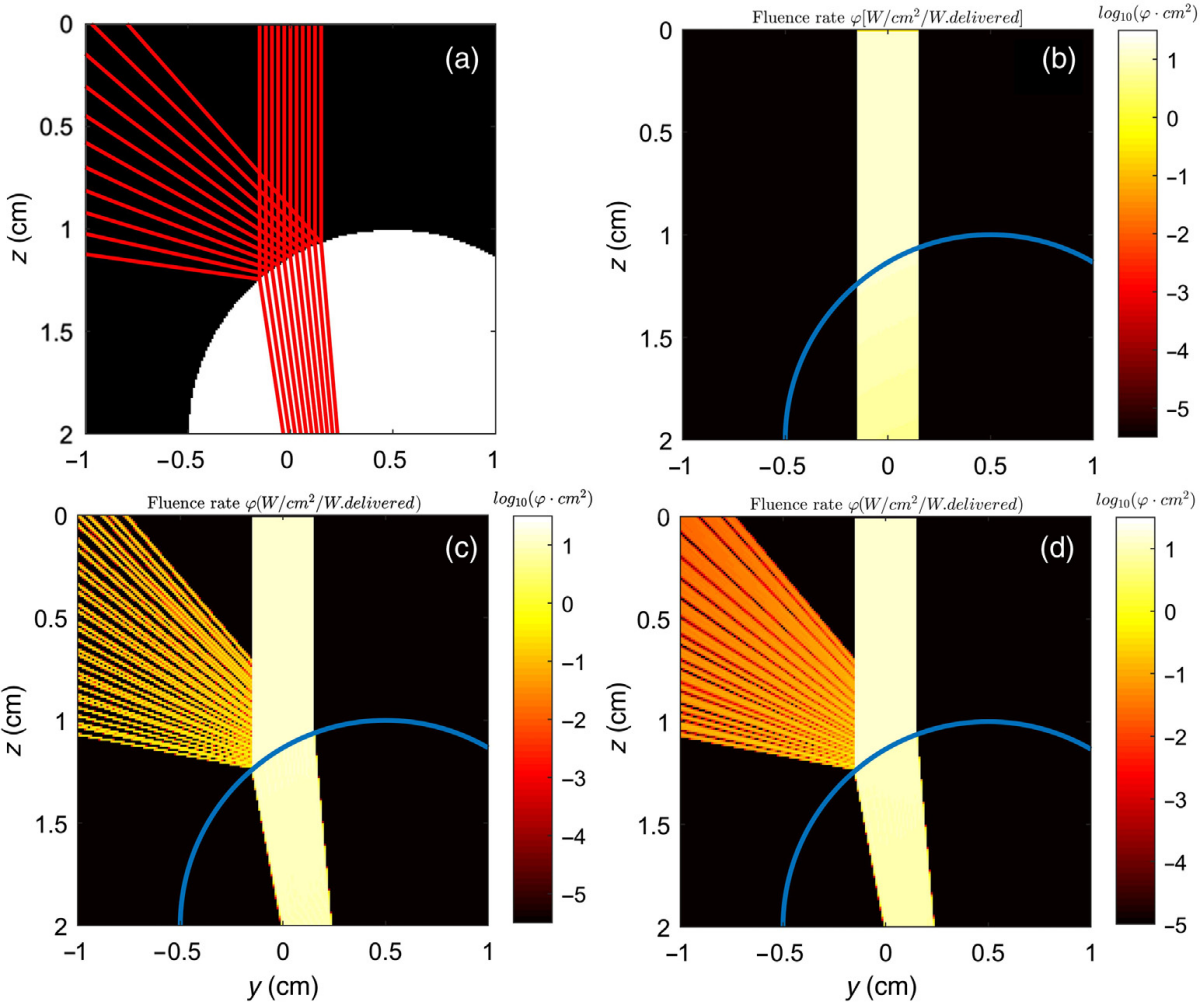
Example of a beam hitting the side surface of a sphere of radius 1 cm. (a) Photon trajectories calculated by Fresnel law, (b) the facet normal method, (c) the surface normal method, and (d) the interpolated surface normal method. The refractivity indices are n=1.00 outside the sphere and n=1.33 inside the sphere. The values of $\mu_{s}$, $\mu_{a}$, and g are picked such that there is no scattering and negligible absorption along the photon paths. The voxel length is 0.01 cm.

In this first example, Fig. 3(a) shows the expected reflected angles from hitting the side surface of a sphere. The facet normal approach in Fig. 3(b) has reflectance directions going back against the laser beam. Here, the error in angle orientation is as high as 80 deg, but the error in reflected angles can be up to 180 deg. The deficiencies of this approach become evident as one deviates from simulating layered models.

On the other hand, the reflected angles in Fig. 3(c) (surface normal method) and Fig. 3(d) (interpolated surface normal method) mimic almost identically the analytical trajectories shown in Fig. 3(a). The differences in reflected and transmitted angles between the analytical solution and the facet normal method are drastic. In contrast, the difference between the analytical solution and that of the surface normal method, interpolated or not, is small, with less than 5 deg error when juxtaposing the edges of the reflected beams. When comparing the interpolated surface normal approach to the simpler surface normal approach, we show that the use of interpolation alleviates almost completely the problem of bunching observed in Fig. 3(c). There are very small gaps observed and that are believed to be due to the approximations made by the proposed approach (as opposed to the detailed approach mentioned in Sec. 2). While the angles are accounted appropriately, the intersections of the photons with mismatched boundary surfaces remain on the faces of the boundary voxels. The observed discontinuities are likely resulting from not performing additional calculations to move the intersections onto the “virtual surface” (illustrated in Fig. 2), a small trade-off in accuracy to preserve fast performances during the simulation.

### 3.2 Lenses

**Fig. 4:**
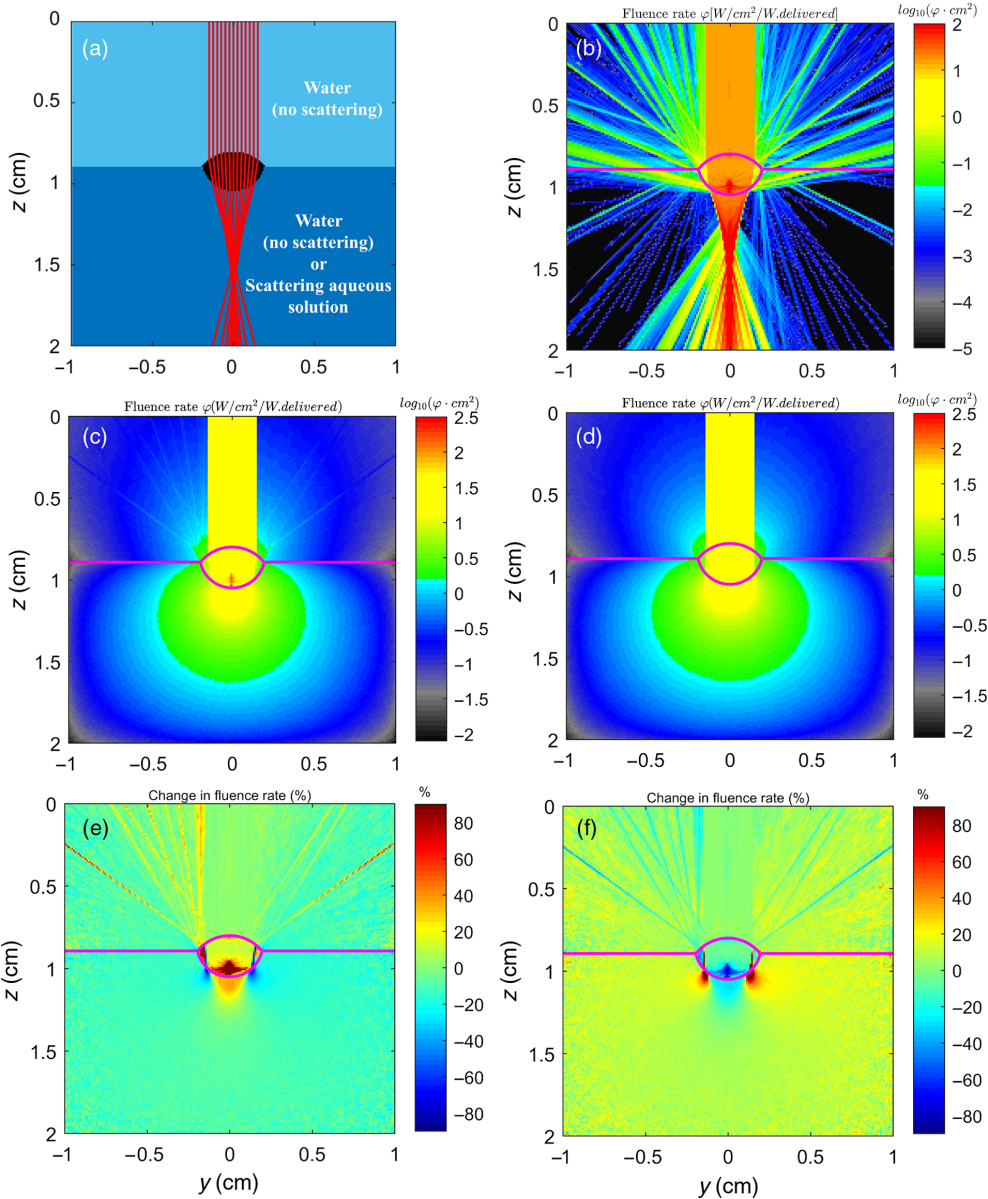
Example of a beam hitting a lens (n=1.52) with the lower half being either water (n=1.33) or aqueous scattering solution (n=1.33, $\mu_{s}=100\;{\mathrm{cm}}^{-1}$, g=0.90) using the interpolated surface normal approach (ISN). (a) Problem setup; (b) lens and water (no scattering) using ISN approach; (c) lens and scattering aqueous solution using ISN approach; (d) lens and scattering aqueous solution using the facet normal (FN) approach; (e) percent change caused by mismatched voxel refractive indices, $\frac{\varphi_{\mathrm{ISN}}-\varphi_{\text{matched}}}{\varphi_{\text{matched}}}\times 100\%$, with scattering present; and (f) percent change caused by using the FN method, as opposed to ISN approach, $\frac{\varphi_{\mathrm{FN}}-\varphi_{\mathrm{ISN}}}{\varphi_{\mathrm{ISN}}}\times 100\%$. The voxel length is 0.01 cm and absorption is assumed to be negligible.

In this second example, a laser beam hitting a lens in a water medium is considered. Figure 4(b) shows that the algorithm is able to specify the focusing of the incoming light by the lens. The algorithm shows excellent agreement with the analytical solution presented in Fig. 4(a). The surface normal approach, either with or without interpolation, adds the ability to properly focus light, which does not occur when using the facet normal approach. Note that the fluence rate inside the lens is increased due to reflectance from the lens surface when using the interpolated surface normal method. The difference in using the facet normal approach as opposed to the interpolated surface normal approach is quite significant. Differences of over 90% in fluence rate are observed near the bottom and the sides of the lens. Large sections of the fluence map also show differences of about 20%.

While it is relatively easy to measure the simulation performance (photons/min), the important alterations that this new, more realistic algorithm has on the photon trajectories make it nontrivial to compare to existing approaches such as the facet normal approach or the simplified approach of ignoring the differences in refractive index. Performance values are reported in Table 1 for the lens examples involving scattering. Speeds (photons/min) were found to be comparable between the surface normal approach and no Fresnel approach. A significant performance drop is not expected going from a facet normal approach to a surface normal approach unless the photon trajectories are significantly altered, which can be the case when dealing with curved and oblique surfaces. This is because the only additional operation that the surface normal approach requires is reading a set of three values indicating the surface normal at the current voxel whenever it encounters a mismatched boundary. The interpolated surface normal approach, on the other hand, drops to about 37% of the speed of its noninterpolated counterpart due to the additional global memory reads and calculations required to perform the interpolation.

**Table 1:** Reported GPU performance for lens simulations.

Simulation type	Simulated photons/min
Scattering and no Fresnel calculations	46.737 millions
Scattering and facet normal approach	46.565 millions
Scattering and surface normal approach	45.761 millions
Scattering and interpolated surface normal approach	16.707 millions

### 3.3 Biological Tissues: Mouse Head

**Fig. 5:**
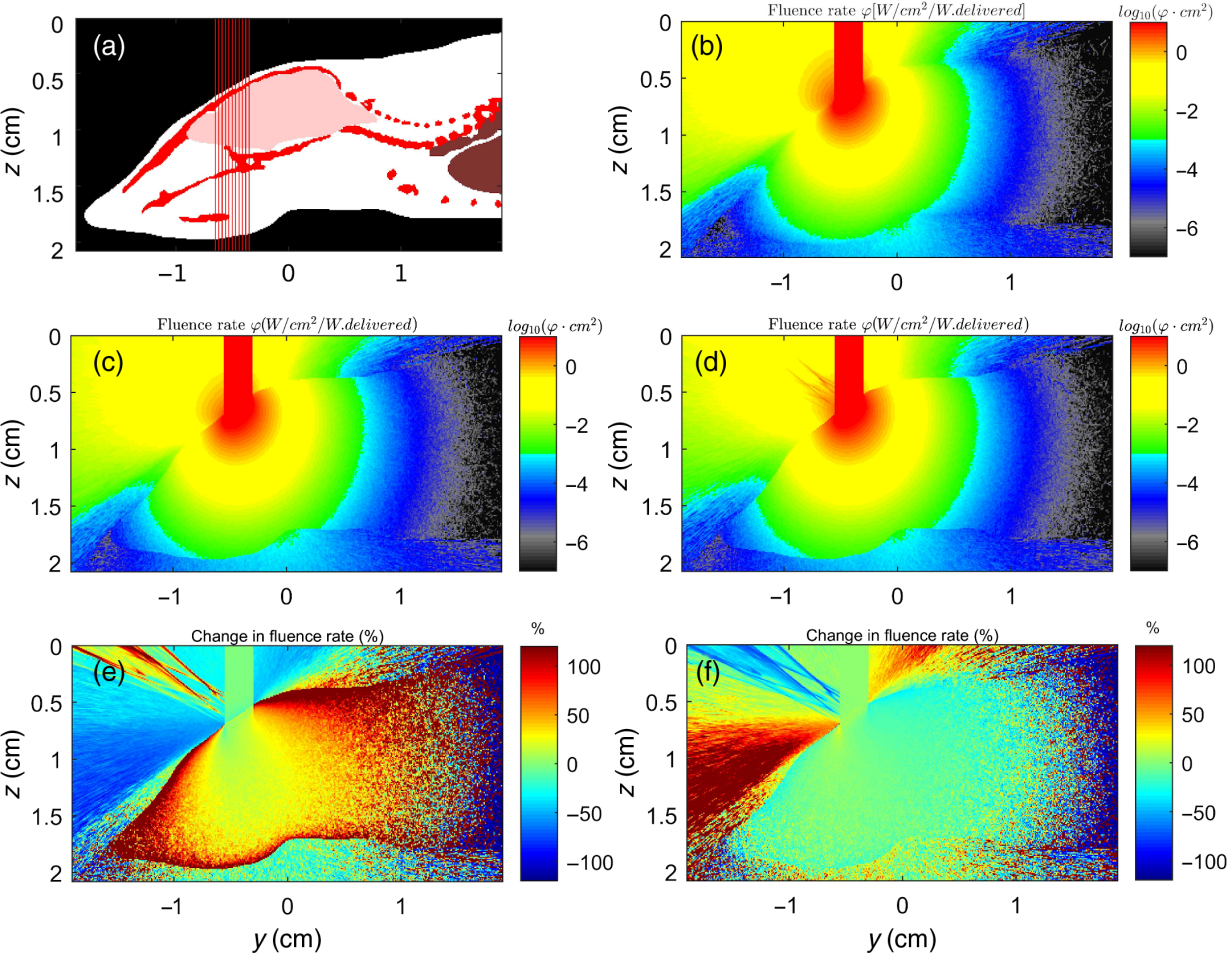
Example of a beam hitting a mouse head. The shape is taken from the Digimouse segmentation.^29^ The inside is simplified to be standard tissue (n=1.33, $\mu_{a}=1\;{\mathrm{cm}}^{-1}$, $\mu_{s}=100\;{\mathrm{cm}}^{-1}$, g=0.90). (a) Problem setup, (b) fluence rate without Fresnel calculations, (c) fluence rate using the facet normal approach, (d) fluence rate using the interpolated surface normal approach, (e) percent change in ϕ caused by mismatched voxel refractive indices, and (f) percent change caused by using the facet normal as opposed to interpolated surface normal approach. The voxel length is 0.01 cm.

The mouse example shown in Fig. 5 represents a typical example of biological tissue simulation, where a beam hits the air/tissue surface boundary at an angle. Figure 5(e) compares the error in the simulation results between using the interpolated surface normal method and the simplified approach that ignores the differences in refractive index. There is a noteworthy weaker overall diffusive reflectance using the interpolated surface normal method as photons that have entered the tissue media have a harder time escaping due to the internal reflection at the mismatched boundary. Also, photons that have entered the tissue remain there longer, leading to a higher fluence rate near the air/tissue boundary. When comparing the interpolated surface normal approach to the facet normal approach, Fig. 5(f) shows that the facet normal handles poorly the reflected fluence and the fluence transmitting out of the mouse body. There are also significant differences between more than 30% in the areas near the inside boundary of the mouse.

## 4 Conclusion

A surface normal approach to light transport is proposed to deal with curved and slanted surfaces when simulating light transport. Across various examples of a beam hitting the side of a sphere, light traversing a lens, and a laser beam hitting the head of a mouse, the proposed methodology leads to significantly more realistic results than the facet normal approach. The core modification to the standard MC simulation was to introduce the use of a gradient map to find a better estimate of the normal vector to the mismatched surface, the use of smoothing on this gradient map, and the application of a trilinear interpolation scheme used to gradually interpolate between various estimates of these calculated normal vectors. Improved accuracy is shown when approximating the expected angles of reflectance and transmittance with a maximum observed error on the reflected angles of less than 5 deg in the case of the sphere example when superposing with the calculated analytical solution. Given the availability of the source code and the fast performances shown on GPU, this approach should be readily applicable to the current state-of-the-art tools, while not introducing a large amount of changes to the MC code. Through this work, an emphasis is made on the importance of dealing with curved and oblique surfaces appropriately along with a way to deal with the problem accurately and efficiently.

## 5 Appendix Detailed Workflow

This section details the steps in Fig. 1 by providing an example of implementation for the core algorithm to calculate the normal vectors in Sec. 5.1 and then expanding on implementation details in Sec. 5.2.

### 5.1 MATLAB Code to Calculate Direction Vectors

%% T is the tissue volume

%% nv is the vector/list of all the refractive indices contained in the tissue list

%% Removing the repeated refractive index values

unique_nv = unique(nv);

%% Creating empty storage maps for the calculated direction vectors

gradient_map_x = zeros(size(T,1),size(T,2),size(T,3));

gradient_map_y = zeros(size(T,1),size(T,2),size(T,3));

gradient_map_z = zeros(size(T,1),size(T,2),size(T,3));

%% Looping through each unique value of refractive index value

for j = 1:length(unique_nv)

 %% Creating 3D binary map for the current unique refractive index value

 n_map = zeros(size(T,1),size(T,2),size(T,3));

 for i = 1:length(n_map(:))

  %% Setting voxel that matches refractive index of the map to 1

  if nv(T(i)) == unique_nv(j)

   n_map(i) = 1;

  end

 end

 %% Finding the direction vectors for the current refractive index value

 [Gx, Gy, Gz] = imgradientxyz(n_map);

 %% Smooth this direction vector map using smoothn

 smooth_map = smoothn({Gx,Gy,Gz},2);

 %% Selectively save the direction vectors contained within the boundaries of each refractive index value domain

 gradient_map_x(n_map == 1) = smooth_map{1}(n_map==1);

 gradient_map_y(n_map == 1) = smooth_map{2}(n_map==1);

 gradient_map_z(n_map == 1) = smooth_map{3}(n_map==1);

end

%% Normalizing final direction vectors

gradient_magn = sqrt(gradient_map_x.^2+gradient_map_y. ^2+gradient_map_z. ^2);

gradient_magn(gradient_magn==0) = 1;

gradient_map_x = gradient_map_x./gradient_magn;

gradient_map_y = gradient_map_y./gradient_magn;

gradient_map_z = gradient_map_z./gradient_magn;

### 5.2 Explanation of the Implementation Details

The numbering in this section follows that of Fig. 1.

#### 5.2.1 Creating a 3D map for the refractive indices

A 3D tissue structure is usually represented by a grid filled with integer or character labels that can be used to look up tissue properties from a tissue list. The function “unique” in MATLAB (or numpy.unique in Python) is applied to the list of refractive index values. This list of unique refractive index (n) values will be used in the second step.

At this stage, there are two possibilities. One possibility is to fill a 3D array with the refractivity index value for each voxel. The other possibility is to use a different label for each of the refractivity index values and create a 3D array with these labels. Either 3D representation works, as only the shapes of the mismatched boundaries are of importance and not the actual magnitudes of the n values.

#### 5.2.2 Creating 3D binary maps for each refractivity index

Going through the list of unique n values (or corresponding labels), a 3D binary map is created for each of the values in this list. Labeling a voxel 1 if it matches the refractivity index or label value of the map and 0 otherwise.

In the illustrated example of Fig. 1, one can start by creating a binary map for n=1.00 or label 1 by looping through each element of the 3D array created in step 1 and perform a Boolean operation to check if the values of the map and voxel correspond (whether it is using the label or refractive index) and assign the appropriate values of 1 if they correspond, 0 if they do not. Then, repeat this step by creating binary maps for n=1.33 and n=1.42.

#### 5.2.3 Applying the Sobel filter to obtain the gradient map

Once the binary maps are calculated in step 2, the function imgradientxyz in MATLAB is applied to these maps to obtain the x, y, and z direction vectors.

This unidirectional Sobel filter provides the direction vectors going away from the mismatched boundary surface within the boundaries defined by the 1 values in the binary maps. This is the orientation that is expected during the calculations of the Fresnel equations. By combining all the maps of direction vectors at the end, the normal vectors on either side of all mismatched boundaries will be reconstituted. This aggregation occurs after the smoothing step.

We note that the standard Sobel filter can also be used similarly with the right modification, the details of which are skipped here.

#### 5.2.4 Smoothing the 3D gradient maps using smoothn and combining the smoothed gradient maps

The next step is to apply smoothn ^25^^,^^26^ to the x,y,z directions calculated by the Sobel filter for each of the labels or refractive index value (an implementation of smoothn in Python is also available). It is recommended that an appropriate smoothing factor is specified rather than using the default value (the smoothing factor equals 2 in the program listed in Sec. 5.1). If this parameter is chosen to be too small, the smoothing will not affect the direction vectors much, while values of this parameter that are taken to be too high will likely lead to significant deformations in the estimated mismatched surface orientations.

Since the objective of this step is to combine all these smoothed maps together, the overlaps between these maps need to be resolved. This can very simply be done using the binary map of each refractivity index (or label) and only saving the direction vectors for which the binary maps indicate values of 1. We refer to the code in Sec. 5.1 for details on how this is done. The alternative way to remove overlaps is what was described in Sec. 2 in which a multiplication of the direction vectors is performed with the corresponding elements on the 3D binary map of the same label or refractive index value, then the sum of all these smoothed map is taken to calculate the aggregate 3D map with all the direction vectors.

The last step of this process is to normalize the direction vectors such that the direction vectors are of length 1. For each voxel, simply calculate the magnitude $\sqrt{x^{2}+y^{2}+z^{2}}$ and divide each x, y, and z components of the direction vectors by this value.

#### 5.2.5 Use of direction vectors during MC simulation and interpolation scheme to calculate intermediate angles

The surface normal method is relatively straightforward as it simply provides the normal angles when photons are attempting to cross a mismatched boundary and the Fresnel equations need to be calculated during the MC simulation.

The adapted trilinear interpolation scheme starts by defining the difference coordinates $x_{d}$, $y_{d}$, and $z_{d}$. These calculations are most easily done assuming that the coordinate system used in propagating the photons is using voxel coordinates. An assumption made is that each calculated direction vector is calculated for the centroid of a given voxel. For example, voxel (0, 0, 0) will contain the corners (0, 0, 0) and (1, 1, 1) and have a centroid of (0.5, 0.5, 0.5). In voxel coordinates, the current position of the photon is defined as ($x_{\mathrm{pos}}$, $y_{\mathrm{pos}}$, $z_{\mathrm{pos}}$). To calculate the difference coordinates, the following equations are used:

$$x_{d}=x_{\mathrm{pos}}-\text{round}(x_{\mathrm{pos}})+0.5,$$ (1)

$$y_{d}=y_{\mathrm{pos}}-\text{round}(y_{\mathrm{pos}})+0.5,$$ (2)

$$z_{d}=z_{\mathrm{pos}}-\text{round}(z_{\mathrm{pos}})+0.5.$$ (3)

When a photon is hitting the mismatched boundary, it is important to know on which side of the boundary the photon is currently located. Instead of interpolating from eight vertices, the vertices of the “interpolation cube” being defined as the centroids of the neighboring voxels, the interpolation scheme presented here only applies to the centroids that have the same refractive index as the current voxel from which the photon is crossing the boundary. Eight Boolean values $v_{xyz}$ are introduced and take the values of 1 for the centroids that have a refractive index that matches the current voxel value and 0 otherwise. We refer to Refs. 27 and 28 to visualize and understand the general workflow of this approach.

The adapted trilinear interpolation equations are then given by

$$c_{00}=c_{000}v_{000}(1-x_{d})+c_{100}v_{100}x_{d},$$ (4)

$$c_{01}=c_{001}v_{001}(1-x_{d})+c_{101}v_{101}x_{d},$$ (5)

$$c_{10}=c_{010}v_{010}(1-x_{d})+c_{110}v_{110}x_{d},$$ (6)

$$c_{11}=c_{011}v_{011}(1-x_{d})+c_{111}v_{111}x_{d},$$ (7)

$$c_{0}=c_{00}(1-y_{d})+c_{10}y_{d},$$ (8)

$$c_{1}=c_{01}(1-y_{d})+c_{11}y_{d},$$ (9)

$$c=c_{0}(1-z_{d})+c_{1}z_{d}.$$ (10)

Here, $c_{xyz}$ represents the values at the vertices and c is the final predicted value using these vertices. This set of equations needs to be applied three times to obtain each of the three components (x,y,z) of the estimated direction vector.

The final step is to normalize this interpolated direction vector and use this direction vector as a normal vector when solving the Fresnel equations.
